# Supplementary figures and images for: Efficiency of Skeletal Muscle Mass/Weight Measurement for Distinguishing Metabolic Dysfunction-Associated Steatotic Liver Disease: A Prospective Analysis Using InBody Bioimpedance Devices
Source: Nutrients. 2024 Dec 23;16(24):4422. doi: 10.3390/nu16244422 (PMC11677209; doi:10.3390/nu16244422)

## Slide 1
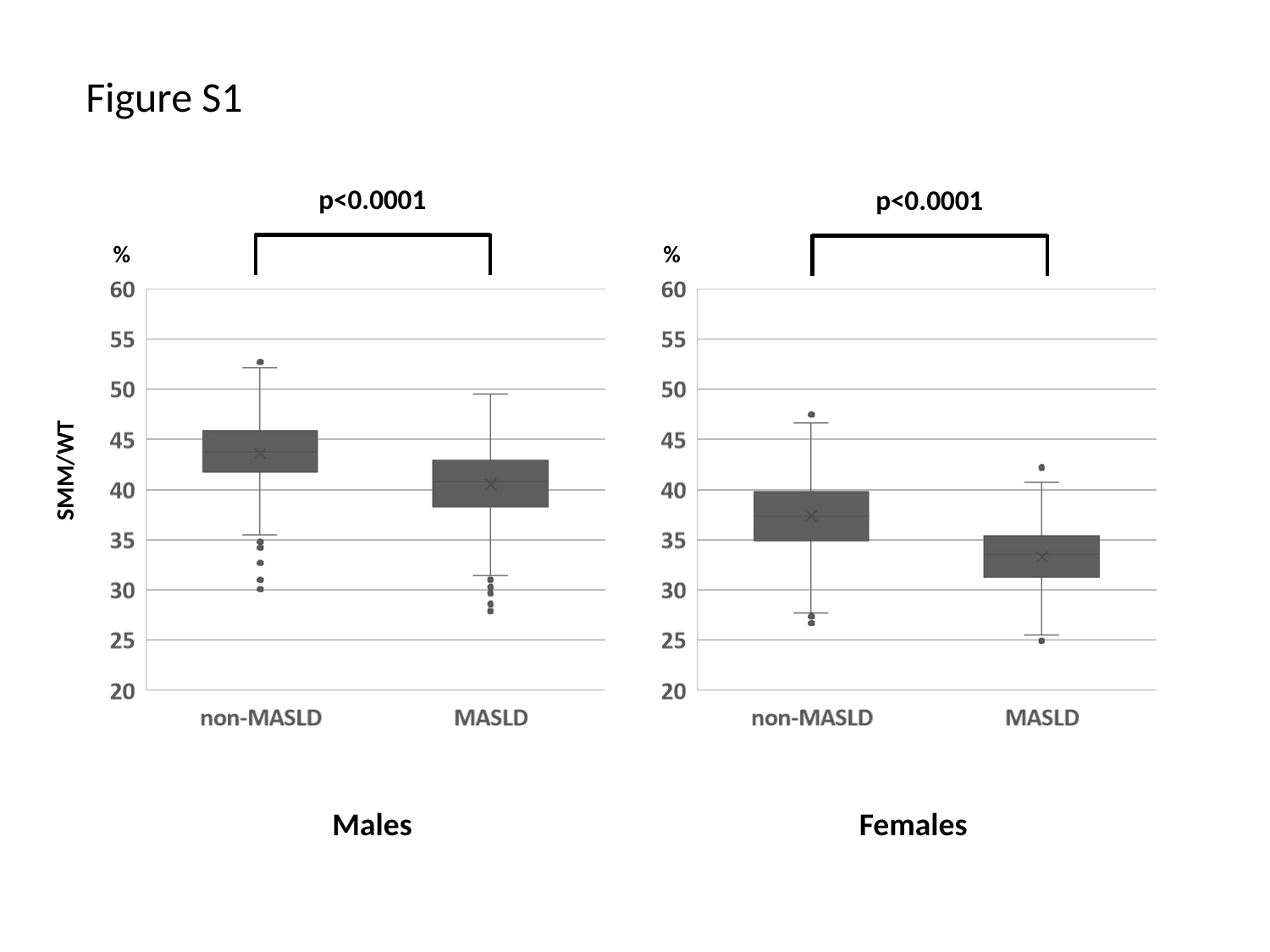

Figure S1
p<0.0001
p<0.0001
%
%
SMM/WT
Females
Males

Supplement: Supplementary file 1 [file nutrients-16-04422-s001.zip › Supplementary Material .pptx]
